# Validating the PVL-Delta model for the Iowa gambling task

**DOI:** 10.3389/fpsyg.2013.00898

**Published:** 2013-12-03

**Authors:** Helen Steingroever, Ruud Wetzels, Eric-Jan Wagenmakers

**Affiliations:** ^1^Psychological Methods, Department of Psychology, University of AmsterdamAmsterdam, Netherlands; ^2^Informatics Institute, University of AmsterdamAmsterdam, Netherlands; ^3^Spinoza Centre for NeuroimagingAmsterdam, Netherlands

**Keywords:** reinforcement learning, expectancy valence model, prospect valence model, test of selective influence, parameter space partitioning

## Abstract

Decision-making deficits in clinical populations are often assessed with the Iowa gambling task (IGT). Performance on this task is driven by latent psychological processes, the assessment of which requires an analysis using cognitive models. Two popular examples of such models are the Expectancy Valence (EV) and Prospect Valence Learning (PVL) models. These models have recently been subjected to sophisticated procedures of model checking, spawning a hybrid version of the EV and PVL models—the PVL-Delta model. In order to test the validity of the PVL-Delta model we present a parameter space partitioning (PSP) study and a test of selective influence. The PSP study allows one to assess the choice patterns that the PVL-Delta model generates across its entire parameter space. The PSP study revealed that the model accounts for empirical choice patterns featuring a preference for the good decks or the decks with infrequent losses; however, the model fails to account for empirical choice patterns featuring a preference for the bad decks. The test of selective influence investigates the effectiveness of experimental manipulations designed to target only a single model parameter. This test showed that the manipulations were successful for all but one parameter. To conclude, despite a few shortcomings, the PVL-Delta model seems to be a better IGT model than the popular EV and PVL models.

## 1. Introduction

The Iowa gambling task (IGT; Bechara et al., 1994) is arguably the most popular neuropsychological paradigm to assess decision-making deficits in clinical populations. In order to isolate and identify the psychological processes that drive performance on the IGT, behavioral analyses of IGT data are insufficient. A promising alternative analysis approach is to use cognitive process models. The IGT imposes high demands on these models because it is a complex task producing various types of choice patterns that a good model should be able to generate (Steingroever et al., 2013a,b). In addition, the models should also account for individual differences and for participants' switch behavior on the task (e.g., Zhao and Costello, 2007; Steingroever et al., 2013b). Despite the high demands, some plausible and elegant IGT models have been proposed. Two of the most frequently used representatives include the Expectancy Valence model (EV; see Steingroever et al., 2013b, for references), and the Prospect Valence Learning model (PVL; see Steingroever et al., 2013b, for references and a detailed description of the models). The parameters of these models correspond to distinct psychological processes such as motivation, learning/memory, and response consistency (Busemeyer et al., in press).

Since the development of the EV model in 2002, reinforcement-learning (RL) models for IGT data have been subjected to sophisticated procedures of model checking (e.g., Busemeyer and Stout, 2002; Yechiam and Busemeyer, 2005; Yechiam and Ert, 2007; Ahn et al., 2008; Yechiam and Busemeyer, 2008; Fridberg et al., 2010; Steingroever et al., 2013b). These model comparison efforts spawned a hybrid version of the EV and PVL models—the PVL-Delta model (Ahn et al., 2008; Fridberg et al., 2010; Steingroever et al., in press; see next section for a detailed description of the PVL-Delta model and recent model comparison efforts). This model seems to be promising for IGT data because it can generate a variety of empirical choice patterns better than its competitors (Steingroever et al., in press).

Whereas previous procedures of model checking focused mostly on relative comparisons of different RL models for IGT data, no efforts have been carried out to validate the PVL-Delta model (i.e., assess its adequacy in isolation). Here, we focus on two different ways of validating the PVL-Delta model: first, we conduct a parameter space partitioning (PSP) study that systematically assesses which choice patterns the PVL-Delta model generates across its entire parameter space. Thus, with this first validity check we aim to answer the question: can the PVL-Delta model generate typical empirical choice patterns over a wide range of parameter settings? Second, we conduct a test of selective influence that investigates the effectiveness of experimental manipulations designed to target only one of the model parameters. Thus, with this second validity check we aim to answer the question: do the parameters of the PVL-Delta model indeed correspond to the proposed psychological processes?

The outline of this article is as follows. In the first section, we explain the IGT, outline the PVL-Delta model, and review previous efforts to compare RL models for IGT data. In the second and third section, we present the PSP study and the test of selective influence. In the last section, we summarize our findings and discuss their ramifications. To anticipate our results, our PSP study shows that the PVL-Delta model can account for empirical choice patterns featuring a preference for the good decks or the decks with infrequent losses; however, the model fails to account for empirical choice patterns featuring a preference for the bad decks. Our test of selective influence shows that the manipulations were successful for all but one parameter.

## 2. The Iowa gambling task and the PVL-Delta model

### 2.1. The Iowa gambling task

In this section we describe the IGT (see also Steingroever et al., 2013b; Steingroever et al., in press). The purpose of the IGT is to measure decision-making deficits of clinical populations in an experimental setting. In the traditional IGT, participants are initially given $2000 facsimile money and are presented with four decks of cards. Participants are instructed to choose cards in order to maximize their long-term net outcome (Bechara et al., 1994, 1997). Unbeknownst to the participants, the task typically contains 100 trials. After each choice, participants receive feedback on the rewards and the losses (if any) associated with that card, and the running tally.

The task aims to determine whether participants learn to prefer the good, safe decks over the bad, risky decks because this is the only choice pattern that maximizes the long-term net outcomes. The good, safe decks are typically labeled C and D, whereas the bad, risky decks are labeled A and B. Table 1 presents the traditional payoff scheme as developed by Bechara et al. (1994). This table illustrates that decks A and B yield high immediate, constant rewards, but even higher unpredictable, occasional losses: hence, the long-term net outcome is negative. Decks C and D, on the other hand, yield low immediate, constant rewards, but even lower unpredictable, occasional losses: hence, the long-term net outcome is positive. In addition to the different payoff magnitudes, the decks also differ in the frequency of losses: two decks yield frequent losses (decks A and C) and two decks yield infrequent losses (decks B and D).

**Table 1:** **Payoff scheme of the traditional IGT as developed by Bechara et al. (1994)**.

	**Deck A**	**Deck B**	**Deck C**	**Deck D**
	**Bad deck with frequent losses**	**Bad deck with infrequent losses**	**Good deck with frequent losses**	**Good deck with infrequent losses**
Reward/trial	100	100	50	50
Number of losses/10 cards	5	1	5	1
Loss/10 cards	−1250	−1250	−250	−250
Net outcome/10 cards	−250	−250	250	250

### 2.2. The PVL-Delta model

In this section, we describe the PVL-Delta model in detail. The model formalizes participants' performance on the IGT through the interaction of four model parameters that represent distinct psychological processes (Ahn et al., 2008; Fridberg et al., 2010; Steingroever et al., in press).

The first model assumption is that after choosing a card from deck *k* ∈ {1, 2, 3, 4} on trial *t*, participants evaluate the net outcome associated with the just-chosen card by means of a non-linear utility function from Prospect theory (Tversky and Kahneman, 1992)—the Prospect Utility function:

(1) $$u_{k}(t)=\{\begin{array}{c} \;X{\left(t\right)}^{A}\text{ if }X(t)\geq 0\\ -w\cdot |X(t){|}^{A}\text{if }X(t)<0. \end{array}$$

Here *X(t)* represents the net outcome on trial *t*, that is, the sum of the experienced reward and loss (i.e., *X*(*t*) = W(t)−|L(t)|). The Prospect Utility function contains the first two model parameters—the shape parameter *A* ∈ [0, 1], that determines the shape of the utility function, and the loss aversion parameter *w* ∈ [0, 5]. As *A* approaches zero, the shape of the utility function approaches a step function. The implication of such a step function is that given a positive net outcome *X*(*t*), all utilities are similar because they approach one, and given a negative net outcome *X*(*t*), all utilities are also similar because they approach −*w*. On the other hand, as *A* approaches one, the subjective utility *u*_*k*_(*t*) increases in direct proportion to the net outcome, *X*(*t*). A value of *w* larger than one indicates a larger impact of negative net outcomes than positive net outcomes on the subjective utility, whereas a value of *w* approaching one indicates identical impact of negative net outcomes and positive net outcomes. As *w* approaches zero, the model predicts that negative net outcomes will be neglected.

The PVL-Delta model further assumes that, after having formed the utility of the just chosen deck through Equation 1, decision makers update their expected utility of the just chosen deck, while keeping the expected utilities of the remaining decks unchanged. This updating process is described by the Delta learning rule:

(2) $$Ev_{k}(t)=Ev_{k}(t-1)+a\cdot (u_{k}(t)-Ev_{k}(t-1)).$$

The Delta learning rule states that the expected utility of the chosen deck *k* is adjusted upward if the experienced utility *u*_*k*_(*t*) is higher than expected. If the experienced utility *u*_*k*_(*t*) is lower than expected, the expected utility of deck *k* is adjusted downward^1^. This updating process is influenced by the third model parameter—the updating parameter *a* ∈ [0, 1]. This parameter quantifies the memory for rewards and losses. A value of *a* close to zero indicates slow forgetting and weak recency effects, whereas a value of *a* close to one indicates rapid forgetting and strong recency effects.

In the next step, the model assumes that the expected utilities of each deck guide participants' choices on the next trial *t*+1. This assumption is formalized by the softmax choice rule, also known as the ratio-of-strength choice rule. The PVL-Delta model uses this rule to compute the probability of choosing each deck on each trial (Luce, 1959; Equation 3). This rule contains a sensitivity parameter θ that indexes the extent to which trial-by-trial choices match the expected deck utilities. Values of θ close to zero indicate random choice behavior (i.e., strong exploration), whereas large values of θ indicate choice behavior that is strongly determined by the expected utilities (i.e., strong exploitation).

(3) $$P[S_{k}(t+1)]=\frac{e^{\theta \cdot Ev_{k}(t)}}{\sum_{j=1}^{4}e^{\theta \cdot Ev_{j}(t)}}$$

The PVL-Delta model assumes a trial-independent sensitivity parameter θ, which depends on the final model parameter: the response consistency *c* ∈ [0, 5] (Equation 4). Small values of *c* cause a random choice pattern, whereas large values of *c* cause a deterministic choice pattern.

(4) $$\theta =3^{c}-1$$

In sum, the PVL-Delta model has four parameters: (1) The shape parameter *A*, which determines the shape of the utility function, (2) the loss aversion parameter *w*, which quantifies the weight of net losses over net rewards, (3) the updating parameter *a*, which determines the memory for past expectancies, and (4) the response consistency parameter *c*, which determines the amount of exploitation vs. exploration.

### 2.3. Previous comparisons of RL models

This section reviews previous model comparison studies. These studies compared the EV model, PVL model, and alternative RL models using a large variety of methods, for instance: the *post hoc* fit criterion (i.e., Busemeyer and Stout, 2002; Yechiam and Busemeyer, 2005; Yechiam and Ert, 2007; Ahn et al., 2008; Yechiam and Busemeyer, 2008; Fridberg et al., 2010),^2^ the simulation method (i.e., Ahn et al., 2008; Fridberg et al., 2010; Steingroever et al., in press; Worthy et al., 2013), tests of generalizability (i.e., Yechiam and Busemeyer, 2005; Yechiam and Ert, 2007; Ahn et al., 2008; Yechiam and Busemeyer, 2008), tests of parameter consistency (i.e., Yechiam and Busemeyer, 2008), and PSP (i.e., Steingroever et al., 2013b)^3^.

The above model comparison studies revealed many positive properties of RL models: first, RL models predict the choices on the next trial better than a Bernoulli baseline model (Busemeyer and Stout, 2002; Yechiam and Busemeyer, 2005; Yechiam and Ert, 2007; Ahn et al., 2008; Yechiam and Busemeyer, 2008, Fridberg et al., 2010)^4^. Second, parameters from the RL models estimated from one RL task can be used to predict performance on a different RL task (Yechiam and Busemeyer, 2005; Yechiam and Ert, 2007; Ahn et al., 2008; Yechiam and Busemeyer, 2008). Third, the loss aversion parameter and the updating parameter of the EV model are stable across different tasks (Yechiam and Busemeyer, 2008). Fourth, the estimated model parameters can be used to improve the prediction of group membership (i.e., chronic cannabis users vs. healthy controls; Fridberg et al., 2010).

These positive properties confirm that cognitive modeling analyses are indeed useful to learn more about the psychological processes that drive performance on the IGT. However, previous model comparison studies also revealed that, even though the EV and PVL models are frequently used, they fail to outperform their competitors consistently. It appears that the performance of the RL models depends on the data set and the method used to assess model performance (i.e., fit performance vs. simulation performance; see Steingroever et al., in press, for a more detailed discussion on previous comparisons of RL models).

Instead of accepting the EV and PVL models as default models to describe IGT data, there is growing evidence that the PVL-Delta model may be a promising alternative IGT model: first, Ahn et al. (2008) showed that the PVL-Delta model results in the best simulation performance (i.e., prediction of the entire sequence of choices on the IGT under a new, unobserved payoff sequence) among the EV model, PVL model, and any combination of the components of the two models. Second, Fridberg et al. (2010) showed that, in two data sets, the PVL-Delta model outperforms the EV model in terms of *post hoc* fit and simulation performance. Third, Steingroever et al., (in press) showed that, among the EV, PVL, and PVL-Delta models, the PVL-Delta model is the only model that adequately generated the choice patterns shown by seven IGT data sets.

Even though the PVL-Delta model has recently come to the fore as a promising model for IGT data, it has not yet been sufficiently validated. Our goal here is to pursue two methods of validating the PVL-Delta model: a PSP study and a test of selective influence.

## 3. Parameter space partitioning

### 3.1. Methods

We performed a PSP study to evaluate the flexibility of the PVL-Delta model (Pitt et al., 2006, Pitt et al., 2008; see also Steingroever et al., 2013b, who performed a PSP study of the EV model, PVL model, and another hybrid model: the EV model with Prospect Utility function). The PSP method systematically assesses the choice patterns predicted by the PVL-Delta model across its entire parameter space. A model is overly flexible when it can generate not only all choice patterns that are observed empirically, but also choice patterns that are logically possible, but never observed. Instead, one should prefer a less flexible, parsimonious model that—ideally—only generates choice patterns that are also frequently observed in experiments (Pitt et al., 2006, 2008).

Note that PSP is a global method (i.e., the full range of parameter values is considered), whereas the other methods that were used to compare RL models are local (i.e., assessment at a particular point in the model's parameter space; for instance, *post hoc* fit criterion, simulation method, tests of generalizability, and tests of parameter consistency). The advantage of global methods is that they enable one to assess the full range of choice patterns a model can generate, whereas the results of local methods always depend on the idiosyncrasies of any single data set (Pitt et al., 2006, 2008).

Pitt et al. (2006) describe a new search algorithm to implement PSP. In our implementation we did not use their sophisticated search algorithm, but followed the conceptual idea of PSP, and used a grid search that works as follows (see also Steingroever et al., 2013b): for each parameter of the PVL-Delta model, we chose 60 values that were equally spaced over the corresponding parameter range. Each combination of these parameter values was used to generate data for 100 synthetic participants completing a 100-trial IGT. For all analyses in this paper, we scaled the traditional payoffs of the IGT as presented in Table 1 by dividing by 100 (cf. Ahn et al., 2011).

The generated data were used to analyze which choice patterns the PVL-Delta model can generate across its entire parameter space. Such analysis naturally requires a definition of choice patterns. Here we used two different definitions—the “broad definition of choice patterns” and the “restricted definition of choice patterns.” These definitions are the same as used by Steingroever et al. (2013b).

#### 3.1.1. Broad definition of choice patterns

The “broad definition of choice patterns” is intended to provide a general idea of which choice patterns the PVL-Delta model can generate. Following Steingroever et al. (2013b), we defined five possible choice patterns: (1) Preference for the good decks over bad decks (i.e., {*C*, *D*} ≻ {*A*, *B*}), (2) preference for the bad decks over good decks (i.e., {*A*, *B*} ≻ {*C*, *D*}), (3) preference for the decks with infrequent losses over decks with frequent losses (i.e., {*B*, *D*} ≻ {*A*, *C*}), (4) preference for the decks with frequent losses over decks with infrequent losses (i.e., {*A*, *C*} ≻ {*B*, *D*}), and (5) remaining choice patterns. For each parameter combination, we computed the proportion of choices from each deck averaged across all 100 trials and all 100 repeated data generations. These average choice proportions were then sorted to determine the generated rank order of deck preferences for each parameter combination. Finally, we computed the proportion of the entire parameter space occupied by each of the defined choice patterns. Even though we defined five possible types of choice patterns, we assume based on the theory underlying the IGT (Bechara et al., 1994, 1997) and our IGT review (Steingroever et al., 2013a) that a good model for IGT data should only generate the first three types of choice patterns.

#### 3.1.2. Restricted definition of choice patterns

Note that the broad definition of choice patterns only considers the rank order of the overall proportions of choices from each deck averaged over 100 repeated data generations with the same parameter combination. This means that it does not matter whether the PVL-Delta model generated, for example, a very strong or a very weak preference for the good decks over the bad decks. Both generated choice patterns are classified as the choice pattern “good decks over bad decks” (i.e., {*C*, *D*} ≻ {*A*, *B*}). To go beyond this coarse classification, we also analyzed the model's behavior when confronted with pronounced deck preferences. To get an indication of pronounced deck preferences shown by healthy participants on the IGT, we used Steingroever et al. (2013b)'s definition of pronounced deck preferences: specifically, Steingroever et al. (2013b) searched their IGT data pool (*N* = 394; Steingroever et al., 2013a) for healthy participants that chose at least 65% cards from either the good decks (i.e., (*C* + *D*) ≥ 0.65), the bad decks (i.e., (*A* + *B*) ≥ 0.65), or the decks with infrequent losses (i.e., (*B* + *D*) ≥ 0.65). By using the 0.65-criterion, Steingroever et al. (2013b) included healthy participants with pronounced deck preferences and excluded healthy participants with random choice behaviors. For each of these three groups, Steingroever et al. (2013b) computed the mean proportions of choices from each deck (as shown in Table 2). For instance, participants classified to the group “pronounced preference for the good decks” chose on average 36 cards from deck C and 40 cards from deck D. Note that 53.6% of all participants in the Steingroever et al. (2013a) data pool showed a pronounced deck preference by making at least 65% choices from the two most preferred decks. This empirical popularity of pronounced deck preferences underscores how important it is that a RL model for the IGT is able to produce such choice patterns.

**Table 2:** **Mean proportions of choices from each deck and mean proportions of switches during the last 50 trials of healthy participants showing a pronounced deck preference [see Table 4 in Steingroever et al. (2013b)]**.

**Choice pattern**	***N***	**Deck A [sd]**	**Deck B [sd]**	**Deck C [sd]**	**Deck D [sd]**	**Switches during the last 50 trials [25%, 75% quantile] (min, max)w**
(C + D) ≥ 0.65	54	0.10 [0.05]	0.14 [0.05]	0.36 [0.17]	0.40 [0.14]	0.35 [0.08, 0.52]
						(0.00, 0.96)
(A + B) ≥ 0.65	18	0.25 [0.07]	0.52 [0.11]	0.11 [0.05]	0.12 [0.06]	0.43 [0.31, 0.58]
						(0.10, 0.86)
(B + D) ≥ 0.65	139	0.12 [0.05]	0.37 [0.12]	0.13 [0.05]	0.39 [0.12]	0.47 [0.28, 0.66]
						(0.02, 1.00)

Healthy participants are selected from the Steingroever et al. (2013a) data pool (*N* = *394*).

Table 2 thus provides an indication of pronounced deck preferences shown by healthy participants on the IGT. We used the mean proportion of choices from these three constructed groups for our second, restricted definition of choice patterns. Specifically, we define a pronounced preference for the good decks as at least 36 and 40 choices from decks C and D, respectively; we define a pronounced preference for the bad decks as at least 25 and 52 choices from decks A and B, respectively; and we define a pronounced preference for the decks with infrequent losses as at least 37 and 39 choices from decks B and D, respectively. Based on our simulations, we then determined the proportion of the parameter space of the PVL-Delta model that produced choice patterns that satisfy this second, restricted definition.

#### 3.1.3. Switch behavior

**Figure 1:**
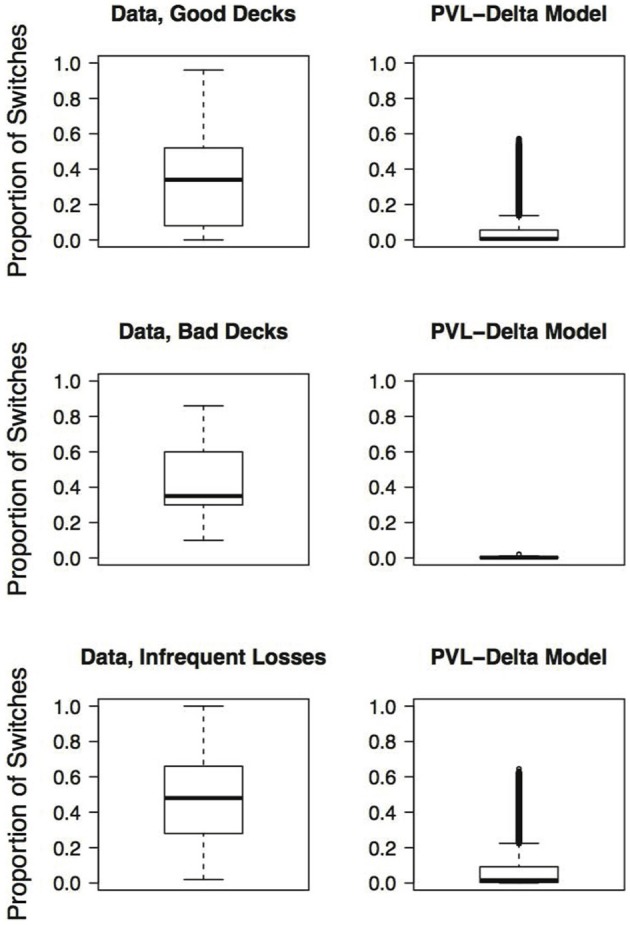
**Boxplots of observed and generated proportions of switches during the last 50 trials, given a pronounced deck preference**. Each row presents the results for different pronounced choice patterns: **First row:** Pronounced preference for the good decks; **Second row:** Pronounced preference for the bad decks; **Third row:** Pronounced preference for the decks with infrequent losses. The first column presents the switches of 211 healthy participants selected from the Steingroever et al. (2013a) data pool (cf. 2). The second column presents the switches generated by the PVL-Delta model (cf. 4).

**Figure 2:**
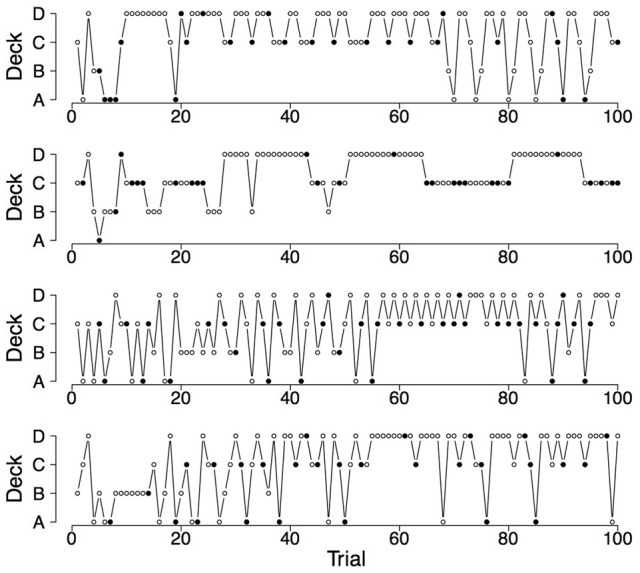
**Deck selection profiles of four healthy participants showing a pronounced preference for the good decks**. The filled dots indicate the occurrence of rewards and losses together; the empty dots indicate the occurrence of only rewards.

**Figure 3:**
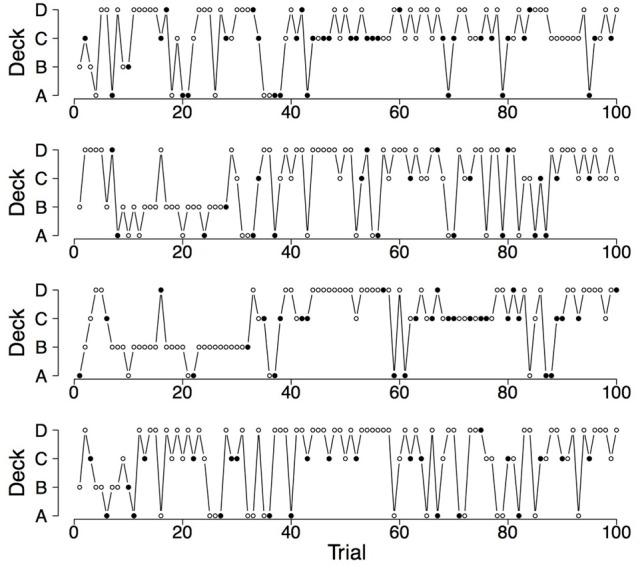
**Deck selection profiles of four synthetic participants showing a pronounced preference for the good decks (generated by the PVL-Delta model; *A* = 0.88, *w* = 0.68, *a* = 0.25, c = 1.27)**.

**Figure 4:**
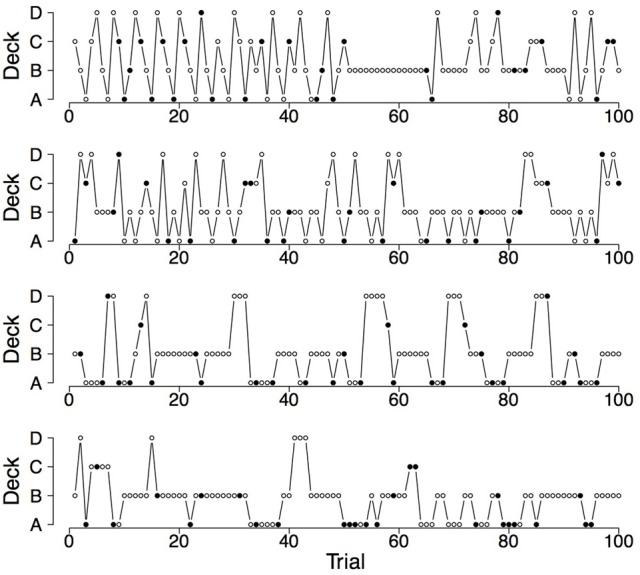
**Deck selection profiles of four healthy participants showing a pronounced preference for the bad decks**.

Finally, a good RL model for the IGT should also capture the switches participants make on the IGT (Zhao and Costello, 2007). Steingroever et al. (2013b) therefore determined the mean proportion of switches during the last 50 trials for the three groups of healthy participants showing pronounced decks preferences (revisited here in the last column of Table 2). The table contains for each of the three groups of healthy participants with pronounced choice patterns the mean proportion of switches during the last 50 trials and statistics quantifying the distribution of switch proportions (i.e., the interquartile range and the minimum and maximum switch proportions during the last 50 trials). This information is visualized by the boxplots shown in the left column of Figure 1. From Table 2 and Figure 1 it is evident that, in general, in all three groups participants switch frequently. However, the interquartile ranges and the minimum and maximum proportion of switches during the last 50 trials also indicate that there is large variability in the proportion of switches, such that the switch behavior of healthy participants varies between no switches at all to switches on every trial. This tendency to switch frequently, but also the large individual differences in the switch behavior of healthy participants are illustrated by Figures 2, 4, 6 (see also Figures 3, 7, 10 in Steingroever et al., 2013b) which show the trial-by-trial choices (i.e., deck selection profiles) of representative healthy participants with a pronounced preference for the good decks, bad decks, and decks with infrequent losses, respectively^5^.

We investigated whether the PVL-Delta model captures the empirical switch behavior by comparing the empirical and generated mean proportions of switches during the last 50 trials. Specifically, the generated mean proportions of switches were obtained by determining the mean proportions of switches during the last 50 trials for all parameter combinations that produced pronounced deck preferences. The code for the PSP study is available on www.helensteingroever.com.

### 3.2. Results

#### 3.2.1. Broad definition of choice patterns

Table 3 presents the proportion of the parameter space of the PVL-Delta model occupied by each of the five different types of choice patterns. From this table, it is evident that the PVL-Delta model can generate all five different types of choice patterns. However, if we consider its partitioned parameter space more closely, we detect substantial differences between the popularity of the different choice patterns: the choice pattern “good decks over bad decks” is the most central to the model's overall performance, as this choice pattern occupies the largest part of the model's parameter space. The second and third largest part of its parameter space are occupied by the choice patterns “remaining” and “infrequent losses over frequent losses.” It is thus evident that choice patterns that are typically shown by healthy participants—the choice patterns “good decks over bad decks” and “infrequent losses over frequent losses” (Steingroever et al., 2013a)—occupy a major part of the model's parameter space.

**Table 3:** **Proportions of choice patterns generated by the PVL-Delta model**.

**Choice pattern**		**Proportion of**
		**all choice patterns**
Good ≻ bad decks	{*C*, *D*} ≻ {*A*, *B*}	0.596
Bad ≻ good decks	{*A*, *B*} ≻ {*C*, *D*}	0.006
Infr. ≻ frequent losses	{*B*, *D*} ≻ {*A*, *C*}	0.118
Frequent ≻ infr. losses	{*A*, *C*} ≻ {*B*, *D*}	0.005
Remaining		0.274

Table 3 also shows that the choice pattern “bad decks over good decks” is only generated over a minor part of the model's parameter space. We have therefore grounds to conclude that this choice pattern is uncharacteristic of the PVL-Delta model, and is thus almost irrelevant to its overall performance (Pitt et al., 2006). This finding is important because the choice pattern “bad decks over good decks” is considered characteristic for participants with decision-making deficits (e.g., patients with lesions to the ventromedial prefrontal cortex; Bechara et al., 1994, 1997). These patients are thought to display decision-making deficits on the IGT because their inability to foresee the long-term consequences of their choice behavior leads them to only focus on the immediate rewards.

#### 3.2.2. Restricted definition of choice patterns

Table 4 presents the proportion of all choice patterns generated by the PVL-Delta model that satisfy the restricted definition of choice patterns. The table also presents the mean and standard deviation of the parameter combinations that generated these pronounced deck preferences. The table shows that only minor parts of the parameter space of the PVL-Delta model are occupied by the three types of pronounced choice patterns, even though these patterns are frequently observed in experiments. For instance, 139 healthy participants from the Steingroever et al. (2013a) data pool (35.3%) show a pronounced preference for the decks with infrequent losses (i.e., (*B* + *D*) ≥ 0.65). However, the PVL-Delta model only generates this choice pattern over 1.6% of its parameter space.

**Table 4:** **Proportion of choice patterns generated by the PVL-Delta model that satisfy the restricted definition of choice patterns**.

**Choice pattern**	**Proportion**	***A* [sd]**	***w* [sd]**	***a* [sd]**	***c* [sd]**	**Switches during**
	**of all choice**					**the last 50 trials**
	**patterns**					**[25%, 75% quantile]**
						**(min, max)**
*C* ≥ 0.36, D ≥ 0.40	0.0084	0.66 [0.21]	0.62 [0.38]	0.30 [0.21]	3.49 [0.98]	0.0571
						[0.0014, 0.0558]
						(0.00, 0.5724)
*A* ≥ 0.25, B ≥ 0.52	0.0000028	0.92 [0.09]	0.02 [0.03]	0.06 [0.03]	3.07 [0.40]	0.0043
						[0.0003, 0.0055]
						(0.00, 0.0210)
*B* ≥ 0.37, D ≥ 0.39	0.0162	0.27 [0.24]	0.34 [0.39]	0.43 [0.27]	2.80 [0.88]	0.0705
						[0.0034, 0.0918]
						(0.00, 0.6450)

Note that this definition is only based on the mean proportion of choices of the two strongest preferred decks (first column). For the selected choice patterns, the corresponding mean and standard deviation of the model parameters, and the mean proportion of switches during the last 50 trials are presented.

#### 3.2.3. Switch behavior

In addition to the generated choice proportions, we also determined the generated proportion of switches during the last 50 trials for all parameter combination that satisfy the restricted definition of choice patterns (Columns 2−6 of Table 4). We averaged these generated switch proportions separately for each of the three types of pronounced deck preferences (last column of Table 4). The table also contains statistics quantifying the distribution of the generated switch proportions, that is, the interquartile range and the minimum and maximum proportion of switches during the last 50 trials. This information is visualized by the right column of Figure 1.

When comparing the generated and observed mean proportion of switches during the last 50 trials given pronounced deck preferences, it is apparent that the PVL-Delta model underestimates the observed switch proportions, that is, the generated mean proportion of switches equals or falls below 0.07 for all generated pronounced choice patterns, whereas the observed mean proportion of switches equals or exceeds 0.35 for all observed pronounced choice patterns (Tables 2, 4). In addition, for all three types of pronounced choice patterns, the interquartile range of the observed proportion of switches exceeds the interquartile range of the model-generated proportion of switches (Figure 1, Tables 2, 4). However, the largest generated switch proportion given a pronounced preference for the good decks and the decks with infrequent losses, respectively, lie within the corresponding interquartile ranges of the observed switch proportions. This suggests that for a few parameter combinations, the PVL-Delta model meets both empirical regularities—pronounced deck preferences and a tendency to switch frequently.

**Figure 5:**
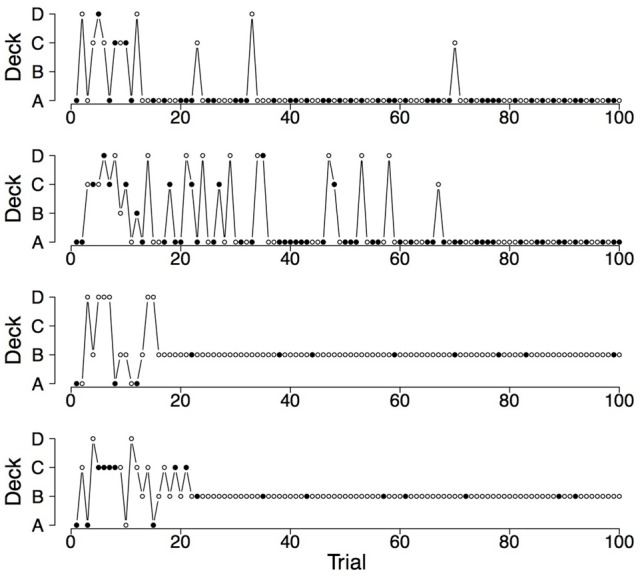
**Deck selection profiles of four synthetic participants showing a pronounced preference for the bad decks (generated by the PVL-Delta model; *A* = 1.00, *w* = 0.08, *a* = 0.05, *c* = 2.71)**.

**Figure 6:**
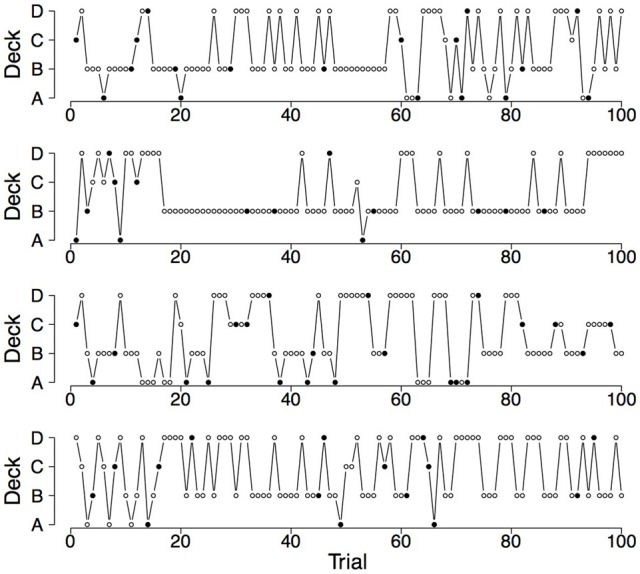
**Deck selection profiles of four healthy participants showing a pronounced preference for the decks with infrequent losses**.

**Figure 7:**
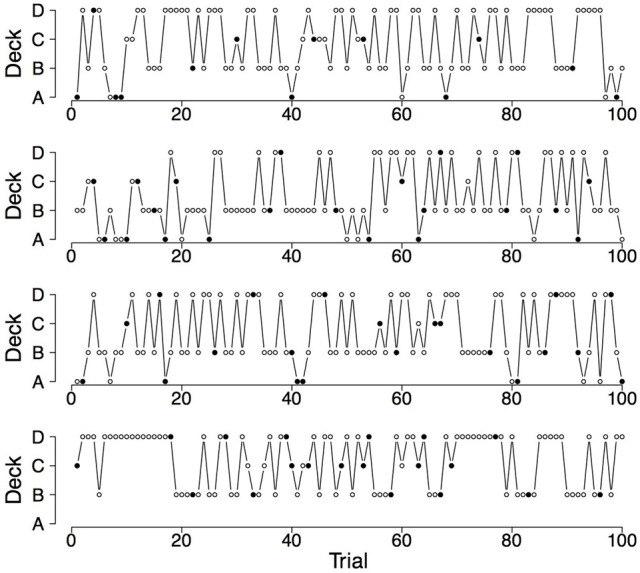
**Deck selection profiles of four synthetic participants showing a pronounced preference for the decks with infrequent losses (generated by the PVL-Delta model; *A* = 0.00, *w* = 0.00, *a* = 0.42, *c* = 1.19)**.

To illustrate the differences and commonalities between the data and the predictions, we plot in Figures 2–7 observed and generated deck selection profiles. Figures 2, 4, 6 show the deck selection profiles of representative healthy participants with a pronounced preference for the good decks, bad decks, and decks with infrequent losses, respectively. Figures 3, 5, 7 show the deck selection profiles that were generated with those parameter combinations that resulted in a pronounced preference for the good decks, bad decks, and decks with infrequent losses, respectively, and the maximum number of switches during the last 50 trials. From the figures it is evident that there are large discrepancies between the observed and generated deck selection profiles in the case of the pronounced preference for the bad decks: The PVL-Delta model generates a few switches in the beginning of the IGT and then exploitation of a single deck, even though healthy participants keep switching across the entire IGT. However, the observed and generated deck selection profiles look very similar in the case of the pronounced preference for the good decks and the decks with infrequent losses.

To conclude, many healthy participants from the Steingroever et al. (2013a) data pool (53.6%) showed pronounced deck preferences, that is, a pronounced preference for the good decks ((*C* + *D*) ≥ 0.65), a pronounced preference for the bad decks ((*A* + *B*) ≥ 0.65), or a pronounced preference for the decks with infrequent losses ((*B* + *D*) ≥ 0.65) (Table 2). This empirical popularity of pronounced deck preferences is only partly reflected by the PVL-Delta model; the model produces choice patterns that satisfy the restricted definition of choice patterns only within minor parts of its parameter space (Table 4). In addition, healthy participants in general show many switches during the last 50 trials. However, the PVL-Delta model in general predicts that participants who show pronounced deck preferences switch rarely during the last 50 trials; all generated mean proportion of switches during the last 50 trials equal or fall below 0.07 whereas the observed mean proportions of switches lie around 0.40. But compared to the popular EV and PVL models (Steingroever et al., 2013b), the PVL-Delta model performs better: the PVL-Delta model generates higher mean proportions of switches than its two competitors for almost all pronounced choice patterns; the only exception is that the EV model generates a higher mean proportion of switches for the choice pattern featuring a pronounced preference for the bad decks than the PVL-Delta model.

Moreover, healthy participants show large individual differences in the proportion of switches during the last 50 trials, such that their switch behavior varies between no switches at all to switches on every trial. However, the PVL-Delta model tends to generate very few switches, given pronounced deck preferences, and fails to generate large proportion of switches (i.e., switch proportions higher than 0.65). But compared to the popular EV and PVL models (Steingroever et al., 2013b), the PVL-Delta model again performs better because the EV and PVL model's failure to generate large proportions of switches, given a pronounced choice pattern, is even stronger: Given a pronounced choice pattern, the EV and PVL models fail to generate switch proportions higher than 0.35 and 0.46, respectively. Despite these discrepancies between the empirical and the generated switch behavior, we showed that—given a pronounced preference for the good decks or the decks with infrequent losses and those parameter combinations that yielded the maximum number of switches during the last 50 trials—the PVL-Delta model can produce choice patterns that strongly resemble the empirical choice patterns of healthy participants.

## 4. Test of selective influence

In this section we investigate whether the parameters of the PVL-Delta model indeed correspond to distinct psychological processes. We will therefore carry out a test of selective influence for the PVL-Delta model. This means that we fit the model to data collected from the standard IGT, but also from conditions that were designed to affect selectively one of the model parameters. These data were collected by Wetzels et al. (2010), and their experiment was originally designed as a test of selective influence for the EV model. However, the experimental manipulations that were intended to affect the parameters of the EV model should also be reflected by the parameters of the PVL-Delta model because of the high similarity between the two models.

### 4.1. Methods

We fit the PVL-Delta model separately to four data sets reported by Wetzels et al. (2010). Specifically, Wetzels et al. (2010) conducted an experiment with a standard condition and three additional conditions that were designed to affect selectively one of the model parameters:^6^ In the “standard condition”, 19 participants completed a 150-trial IGT under the standard administration. In the “rewards condition”, 20 participants completed a 150-trial IGT under the instruction to pay more attention to rewards and to consider losses as less important. We expected this manipulation to decrease the loss aversion parameter *w*.

In the “updating condition”, 19 participants completed a 150-trial IGT under the standard administration. However, each choice was followed by a on-screen presentation of five numbers that the participants had to remember because, after the next choice, participants were asked about the relative position of one of the numbers. We expected this manipulation to increase the updating parameter *a*.

In the “consistency condition”, 16 participants completed a 150-trial IGT under the standard administration. However, they were told that after every 10 trials the payoff schemes for the decks could have changed. We expected this manipulation to decrease the consistency parameter *c*.

To fit the PVL-Delta model, we used a Bayesian hierarchical approach detailed in the next section. This estimation procedure has been consistently shown to outperform alternatives such as maximum likelihood estimation and Bayesian individual estimation (Ahn et al., 2011; Wetzels et al., 2010).

To assess whether the chains of all parameters had converged successfully from their starting values to their stationary distributions, we visually inspected the Hamiltonian Monte Carlo (HMC) chains and used the $\hat{R}$ statistic (Gelman and Rubin, 1992). The $\hat{R}$ statistic is a formal diagnostic measure of convergence that compares the between-chain variability to the within-chain variability. Values close to 1.0 indicate convergence to the stationary distribution, whereas values greater than 1.1 indicate inadequate convergence.

To assess model performance in absolute terms, we used two different methods: the *post hoc* absolute fit method and the simulation method (see also Steingroever et al., in press). These two methods allow us to assess the model's ability to fit and generate the choice patterns present in each of the four conditions. Our implementation of both methods relies on visually contrasting—separately for each deck as a function of 15 bins each containing 10 trials—the observed mean choice proportions from the experiment against the mean choice probabilities from the model.

Both methods start by sampling parameter values from the joint posterior distributions over the individual-level parameters (hereafter individual-level joint posteriors). In the case of the *post hoc* absolute fit method, the model is provided with the sampled parameter values, but also with the actual choices and payoffs of each participant. The *post hoc* absolute fit method computes the probability of choosing each deck on the next trial based on the information on the observed choices and payoffs up to and including the current trial. The simulation method, on the other hand, is only provided with the sampled parameter values, and relies on generating choices for another sequence of payoffs that could have been observed^7^. In particular, on each trial, the simulation method generates a choice based on the predicted choice probabilities. For both methods and for each participant, we repeated the process of obtaining the predicted choice probabilities 100 times to account for uncertainty in the individual-level joint posteriors (for detailed recipes see Steingroever et al., in press)^8^.

To investigate the effect of the experimental manipulations, we visually compared the posterior distributions of the group-level parameters of all four conditions.

#### 4.1.1. Bayesian hierarchical estimation procedure

**Figure 8:**
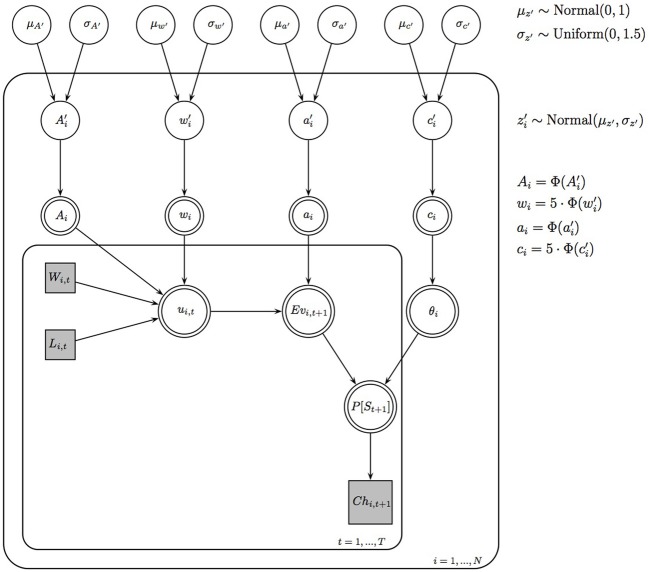
**Bayesian graphical PVL-Delta model for a hierarchical analysis**. Φ() is the cumulative standard normal distribution function.

To fit the PVL-Delta model to the data of the four experimental conditions, we used a Bayesian hierarchical estimation procedure (see Wetzels et al. (2010) for the same model specification in the case of the EV model). The Bayesian graphical PVL-Delta model for a hierarchical analysis is shown in Figure 8. This figure shows that the graphical model consists of two plates: The inner plate expresses the replications of the choices on *t* = 1, …, T trials of the IGT, and the outer plate expresses the replications for *i* = 1,…, N participants. For the sake of clarity, we omitted the notation that indexes the deck number *k*. The quantities *W*_*i*, *t*_ (rewards of participant *i* on trial *t*), *L*_*i*, *t*_ (losses of participant *i* on trial *t*), and *Ch*_*i*, *t* + 1_ (choice of participant *i* on trial *t* + 1) can directly be obtained from the data. The quantities *u*_*i*, *t*_, *Ev*_*i*, *t* + 1_, and θ_i_ are deterministic because they can be calculated from Equations 1, 2, and 4. All individual-level parameters *z*_*i*_, that is, {A_*i*_, w_*i*_, a_*i*_, c_*i*_}, are also deterministic because instead of modeling the individual-level parameters directly, we modeled their respective probit transformations *z*_*i*_', that is, *{A*_*i*_', w_*i*_', a_*i*_', c_*i*_'}. This means that the parameters *z*_*i*_' lie on the probit scale covering the entire real line. The probit transformation is the inverse of the cumulative standard normal distribution function. The parameters *z*_*i*_' are assumed to be drawn from group-level normal distributions with mean μ_z'_ and standard deviation σ_z'_. Only after the analysis was complete, we transformed the parameters μ_z'_ and *z*_*i*_' back to the original scale.

The model specification requires a definition of priors for the group-level means and standard deviations. We assigned a normal prior to the group-level means, μ_z'_ ~ N(0, 1), and a uniform prior to the group-level standard deviations, σ_z'_ ~ U(0, 1.5).

We implemented the PVL-Delta model in Stan (Hoffman and Gelman, 2011;, Stan Development Team, 2013a,b). The code to fit the PVL-Delta model in Stan is available on http://www.helensteingroever.com. To confirm that we correctly implemented the PVL-Delta model, we ran several parameter-recovery studies. The results of two such studies are presented in the Appendix.

For each parameter, we ran three HMC chains simultaneously. The fitting procedure consisted of two steps: First, we initialized all chains with randomly generated starting values. We collected 1000 samples of each chain after having discarded the first 9000 samples of each chain as burn-in. However, this procedure did not result in successful convergence of the HMC chains of all parameters: for instance, for some parameters, two chains may appear to have converged to their stationary distributions and looked like “hairy caterpillars” that are randomly intermixed, whereas the third chain behaved differently and producing an inferior goodness of fit (GOF). Therefore, in a second step, we again ran three HCM chains for each parameter, but this time, we initialized all chains with parameter values close to the mean of the HCM chain that produced the best GOF in the first step. However, even this procedure resulted in convergence problems for a few participants (e.g., bimodal posterior distributions). We therefore excluded participants with such convergence issues and repeated the first and second step. This explains why the sample sizes presented in Table 5 are slightly smaller than those reported by Wetzels et al. (2010).

**Table 5:** **Sample size of the four data sets and number of burn-in samples and posterior samples that we collected for each chain**.

**Experimental**	**Sample**	**Burn-in**	**Posterior**
**condition**	**size**	**samples**	**samples**
Standard	17	37,000	3000
Rewards	19	30,000	3000
Update	16	23,000	1500
Consistency	15	18,000	2000

Table 5 also presents, for each data set separately, the number of burn-in samples and posterior samples that we collected for each chain. These specifications differ across data sets to ensure that all chains reached convergence. We based our inferences on these posterior samples.

### 4.2. Results

In this section, we discuss the results of the test of selective influence. We first focus on the behavioral level by describing the choice patterns observed in the four experimental conditions. Second, we focus on the level of the cognitive modeling analyses; we describe tests confirming that the posterior distributions converged successfully from their starting values to their stationary distributions. In addition, we show that the PVL-Delta model results in a satisfactory fit performance and simulation performance for the four conditions. Finally, we visually compare the posterior distributions of the group-level parameters of all four conditions to draw inferences about the effect of the experimental manipulations.

#### 4.2.1. Behavioral data

**Figure 9:**
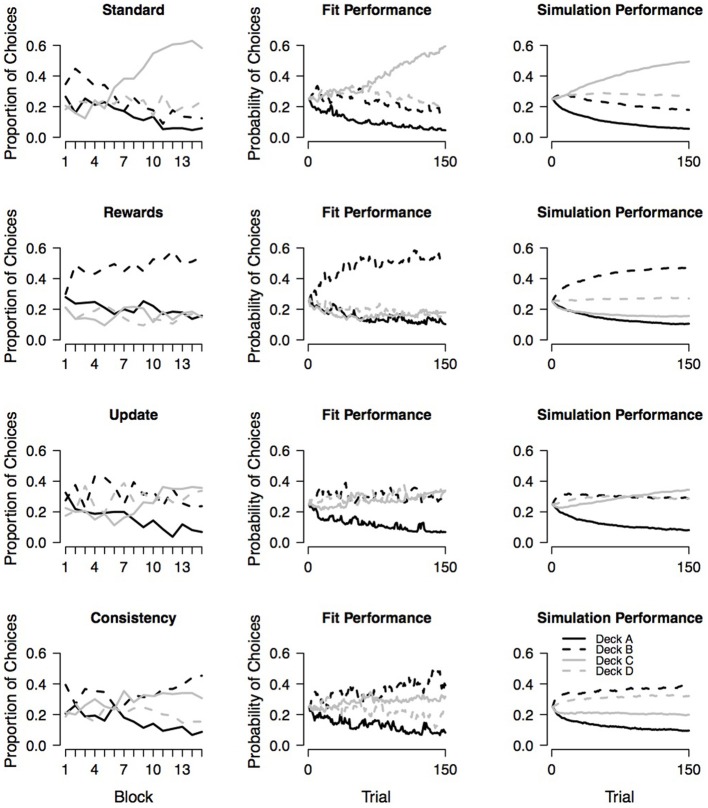
**Observed choice behavior and assessment of absolute model performance**. The first column shows the mean proportion of choices from each deck within 15 blocks as observed in the four experimental conditions reported by Wetzels et al., (2010). Each block contains 10 trials. The second and third column show the fit performance and simulation performance, respectively, for each of the four conditions. Fit performance and simulation performance are based on random draws from the individual-level joint posteriors.

The mean proportion of choices from each deck within 15 blocks each containing 10 trials as observed in the four experimental conditions reported by Wetzels et al. (2010) are presented in the first column of Figure 9. In the standard condition, participants learned to prefer good deck C over all remaining decks; however, participants failed to learn that deck D is also a good deck.

In the rewards condition (i.e., participants were instructed to pay more attention to rewards and to consider losses as less important), participants learned to prefer bad deck B over all remaining decks. Note that even though bad decks A and B both yield high immediate rewards on every trial, participants did not learn to select deck A more often than good decks C and D. This may suggest that the experimental manipulation was only partly successful.

In the updating condition (i.e., each choice was followed by a on-screen presentation of five numbers that participants had to remember because, after the next choice, they were asked about the relative position of one of the numbers), participants show a very weak learning curve; they only learned to avoid deck A.

In the consistency condition (i.e., participants were told that after every 10 trials the payoff schemes for the decks could have changed), participants—in contrast to the intention of the experimental manipulation—did not evenly explore all decks across the entire 100 trials. Instead participants learned to prefer decks B and C over the remaining decks. It seems that participants prefer bad deck B because it yields high immediate rewards on the majority of the trials; however, participants prefer good deck C because it never yields a net loss and is therefore a safe option.

#### 4.2.2. Convergence checks

Visual inspection of the HMC chains and consideration of the $\hat{R}$ statistics for all parameters (all parameters had $\hat{R}$ values below 1.045) suggest that all chains have converged successfully. To illustrate how we visually assessed convergence, we show the chains of one individual-level parameter in the Appendix. From the figure it is evident that the chains have converged successfully from their starting values to their stationary distribution, looking like “hairy caterpillars” that are randomly intermixed.

#### 4.2.3. Absolute model performance

To assess the absolute model performance of the PVL-Delta model with respect to the four experimental conditions, the second and third column of Figure 9 show the fit performance and simulation performance, respectively. Fit performance and simulation performance are based on random draws from the individual-level joint posterior. From the second column of the figure it is evident that the PVL-Delta model provides a good fit to the data of all four conditions (i.e., the model makes accurate one-step-ahead predictions when provided with access to the observed sequence of choices and payoffs). In addition, the third column of Figure 9 illustrates that the PVL-Delta model adequately generates the choice pattern shown by the standard and update conditions. In the case of the rewards and consistency conditions, the simulation performance of the PVL-Delta model is acceptable; the model correctly predicts the most preferred deck, but fails to account for the rank order of the remaining three decks: in the reward condition, the model predicts that deck D is preferred over decks A and C even though the participants chose these three decks about equally often. In the consistency condition, the model predicts that deck D is preferred over deck C even though the participants showed the reverse pattern. To sum up, the PVL-Delta model captures the global patterns in the data providing an acceptable fit and simulation performance with respect to the four data sets at hand; this allows us to meaningfully compare the group-level parameters of the four conditions.

#### 4.2.4. Test of selective influence

**Figure 10:**
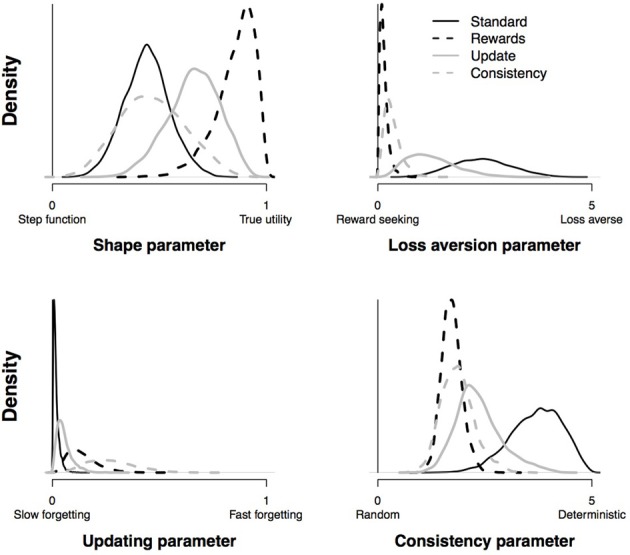
**Posterior distributions for the group-level parameters of the PVL-Delta model in the four experimental conditions**.

Figure 10 presents the posterior distributions of the group-level parameters of all four conditions. It is evident that the experimental manipulation is successfully reflected in the loss aversion parameter and the consistency parameter: first, compared to participants that received the standard instruction, participants who were instructed to focus on rewards (i.e., the rewards condition) had lower values for the loss aversion parameter indicating that they were indeed more reward-seeking. Second, fitting the PVL-Delta model to data of participants that were told that after every 10 trials the payoff schemes for the decks could have changed (i.e., the consistency condition) resulted in a smaller consistency parameter (i.e., a more random choice behavior) than fitting the PVL-Delta model to data of participants that received the standard instructions. However, in the update condition is no clear effect on the updating parameter. Yet, it is evident that the consistency parameter in the update condition is noticeably lower than in the standard condition (i.e., a more random choice behavior); this is consistent with the choice pattern shown by the update condition; participants only learned to avoid deck A, but show a completely indistinguishable preference for the remaining three decks.

## 5. Discussion

In this article, we conducted two tests to validate the PVL-Delta model: a parameter space partitioning study and a test of selective influence. Applying PSP to the PVL-Delta model, we have obtained a deeper understanding of the model's behavior. We used two different definitions of choice patterns; the broad definition allowed us to get an indication of how central each of the choice patterns are to the model's overall performance, and the restricted definition allowed us to assess the model's data-fitting potential when confronted with data featuring pronounced deck preferences.

Using the broad definition of choice patterns, the PSP study revealed that the PVL-Delta model can generate all typical empirical choice patterns. However, the PVL-Delta model generates the choice pattern featuring a preference for the bad decks only over a minor part of its parameter space suggesting that this choice pattern is virtually irrelevant to the model's overall performance.

Using the restricted definition of choice patterns, the PSP study revealed that the PVL-Delta model can still generate all pronounced empirical choice patterns over a minor part of its parameter space. But for these pronounced choice patterns, the PVL-Delta model generally underestimates the empirical switch proportions during the last 50 trials. In particular, given pronounced preferences for the bad decks, the PVL-Delta model fails to account for the empirical switch behavior. This failure seems to be caused by the Prospect Utility function of the PVL-Delta model: in a previous PSP study, Steingroever et al. 2013b showed that this failure is also present in the PVL and EV-PU model (i.e., models with the Prospect Utility function), but not in the EV model (i.e., a model without the Prospect Utility function). However, in the case of the other two pronounced choice patterns—the choice patterns favoring decks with high expected value or low loss frequency—we showed that the PVL-Delta model provides a good account for the empirical switch behavior for some parameter combinations.

The results of the PSP study for the PVL-Delta model and the earlier PSP studies for the EV and PVL models (Steingroever et al., 2013b) suggest that the PVL-Delta model outperforms its two competitors. The EV model fails to generate a pronounced preference for the decks with infrequent losses; the PVL model is able to generate pronounced decks preferences, but underestimates the switch proportions even more strongly than the PVL-Delta model. This superiority of the PVL-Delta model is in line with the posterior predictive checks reported by Steingroever et al., (in press).

An important advantage of PSP is that it is a global analysis technique augmenting local methods that have previously been used to compare RL models (Pitt et al., 2006, 2008). Whereas local methods, such as the *post hoc* fit criterion or the generalization criterion, evaluate a model's performance at a single point of a model's parameter space, global methods such as PSP help us to determine the full range of choice patterns that a model can generate by varying its parameter values (see also Vanpaemel, 2009). This means that we can obtain a global perspective on the data-fitting potential of the PVL-Delta model. Thus, if researches wish to apply the PVL-Delta model to IGT data, they can decide based on the behavioral results whether it is appropriate to apply the PVL-Delta model or not.

The PSP results of this paper should be interpreted with care. PSP gives an indication of how central choice patterns are to the overall performance of the model. However, it is premature to conclude that the PVL-Delta model cannot generate the choice pattern “bad decks over goods decks” at all, soley because the model generates this choice pattern over a small part of the parameter space. Instead, we can only conclude that this choice pattern is not central to the model's overall performance.

It should also be noted that the inferences drawn from the PSP study strongly depend on our definitions of choice patterns. The restricted definition of choice patterns was based on IGT performance of healthy participants (Steingroever et al., 2013b). We could thus detect inconsistencies between the empirical popularity of each pronounced choice pattern in the Steingroever et al. (2013a) data pool and the frequency predicted by the PVL-Delta model. It is troubling that the PVL-Delta model fails to generate a pronounced preference for the bad decks with many switches. But it should be acknowledged that this choice pattern is not central in healthy participants' IGT performance: in the Steingroever et al. (2013a) data pool, only 5% (*N* = 18) of the healthy participants showed this choice pattern (Table 2). Still, this choice pattern is assumed to be characteristic for patients with decision-making deficits (Bechara et al., 1994, 1997), but a better empirical foundation (e.g., a literature review on the IGT performance of clinical groups) is required to accurately judge the gravity of the PVL-Delta model's failure to generate a pronounced preference for the bad decks with many switches.

The test of selective influence revealed that the experimental manipulations had a noticeable effect on the loss aversion parameter and consistency parameter, but not on the updating parameter. However, it is premature to conclude that the updating parameter does not correspond to memory processes. It may be that the experimental manipulation did not work out properly. In addition, one should bear in mind that every data set is characterized by its own idiosyncrasies. IGT data generally are highly idiosyncratic—possibly because the IGT is a very complex task (Steingroever et al., 2013a). In order to be able to draw more accurate conclusions on whether the parameters represent distinct psychological processes, independent repetitions of the test of selective influence and even different experimental manipulations are necessary.

The results of this article confirm that the PVL-Delta model is an attractive alternative to the popular EV and PVL models. However, the PVL-Delta model is also characterized by a few shortcomings because it underrepresents the choice pattern featuring a preference for the bad decks. Nevertheless, we recommend that researchers use the PVL-Delta model to disentangle psychological processes underlying IGT performance, provided that they rigorously assess absolute model performance before interpreting the model parameters (Steingroever et al., in press).

### Conflict of interest statement

The authors declare that the research was conducted in the absence of any commercial or financial relationships that could be construed as a potential conflict of interest.

## Figures and Tables

**Figure A1:**
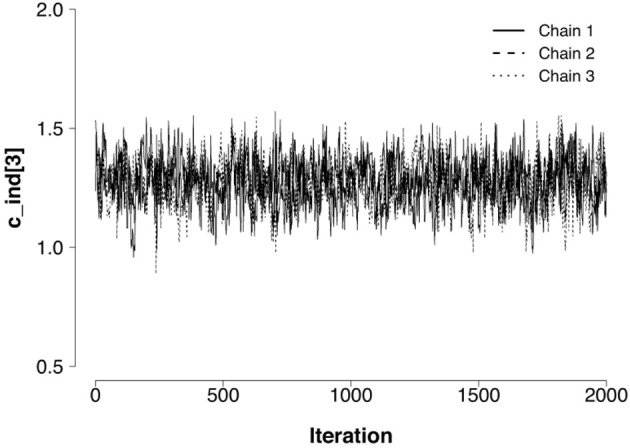
**HCM chains of the individual-level consistency parameter *c* of the third participant in the consistency condition**. In addition to the formal diagnostic measure of convergence $\hat{R}$, we inspected this type of plot for every parameter to visually assess convergence.

**Figure A2:**
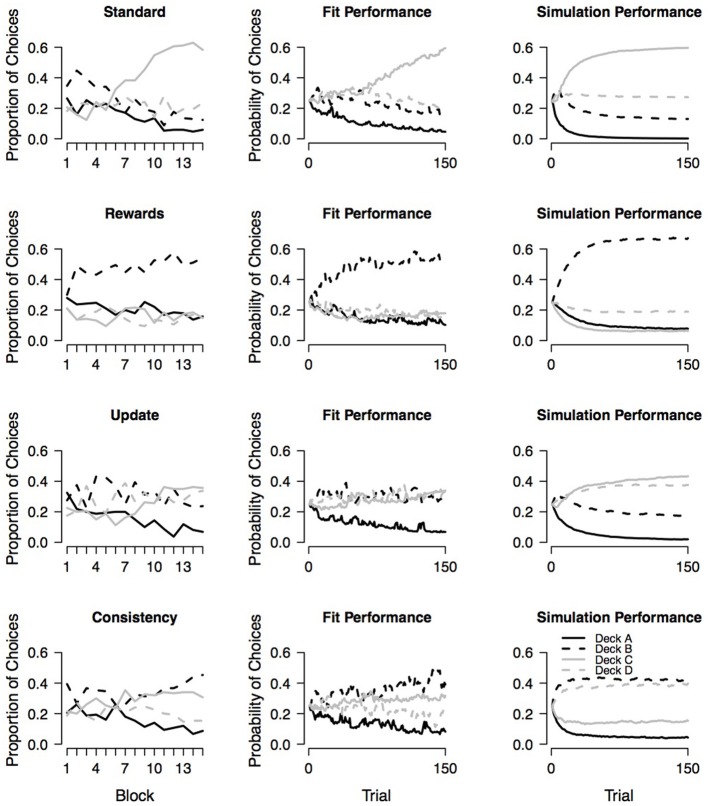
**Observed choice behavior and assessment of absolute model performance**. The first column shows the mean proportion of choices from each deck within 15 blocks as observed in the four experimental conditions reported by Wetzels et al. (2010). Each block contains 10 trials. The second and third column show the fit performance and simulation performance, respectively, for each of the four conditions. Fit performance and simulation performance are based on random draws from the Fit performance and simulation performance are based -level joint posteriors.

**Figure A3:**
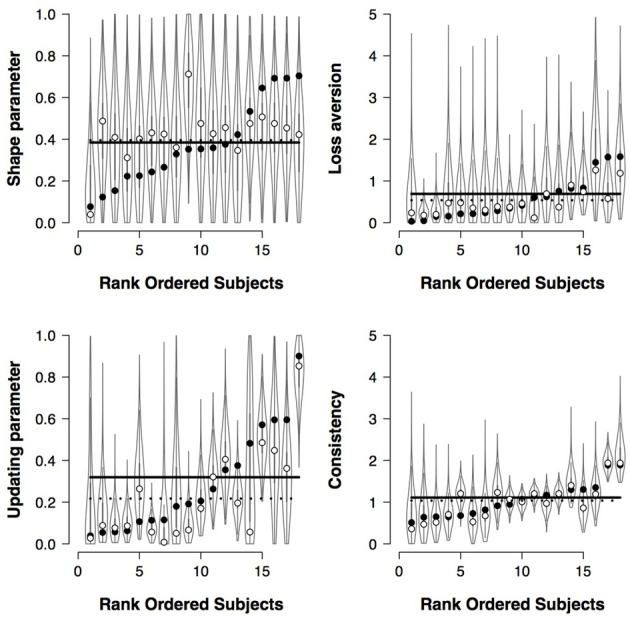
**Recovery of individual and group-level parameters of the PVL-Delta model**. Data of 18 participants completing a 100-trial IGT. The dotted lines represent the modes of the group-level posteriors and the unfilled dots the modes of the group-level posterior.

**Figure A4:**
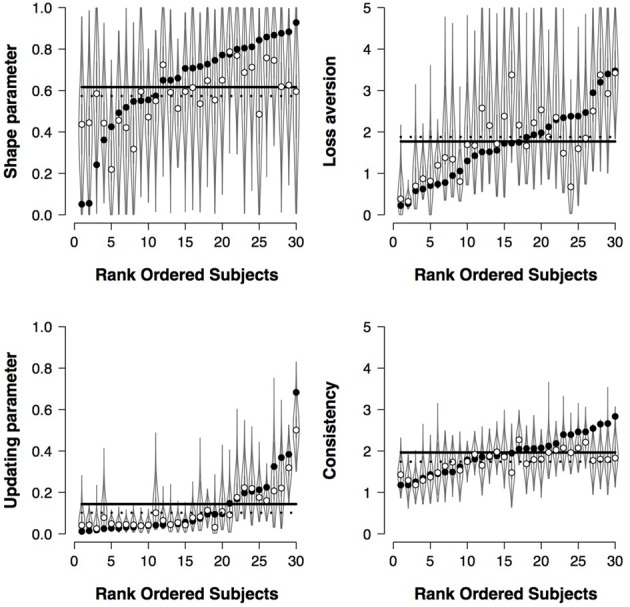
**Recovery of individual and group-level parameters of the PVL-Delta model**. Data of 30 participants completing a 100-trial IGT. The dotted lines represent the modes of the group-level posteriors and the unfilled dots the modes of the individual-level posteriors.

## Footnotes

^1^We initialized the expectancies of each deck *k* to zero, *E*_*vk*_(0) = 0.

^2^The *post hoc* fit criterion is also known as the one-step-ahead prediction method.

^3^Note that the PSP study of Steingroever et al. (2013b) did not focus on the PVL-Delta model, but on the EV model, the PVL model, and another hybrid model: the EV model with the Prospect Utility function.

^4^The Bernoulli baseline model assumes that a participant's probability of choosing a given deck on a given trial equals the overall proportion of choices the participant actually made from that deck.

^5^See Steingroever et al. (2013b) for the deck selection profiles of all healthy participants that showed a pronounced deck preference (i.e., at least 65% choices from the two most preferred decks).

^6^Note that we use the data sets that Wetzels et al. (2010) obtained after having eliminated two sources of contamination. Specifically, Wetzels et al. (2010) removed participants for whom one or more of the maximum likelihood point estimates were located on the boundary of the parameter space, and participants for whom the Bernoulli baseline model outperformed the EV model.

^7^Note that we used the same payoff schedule as in the corresponding experiment.

^8^For completeness, we also produced predicted choices based on the joint posterior of the group-level parameters (hereafter group-level joint posterior); that is, we generated data with 1000 parameter values that were randomly drawn from the group-level joint posterior. There are slight differences between the two types of posterior predictives, but the general conclusions are the same (see Appendix for further details).

## Appendix

In this appendix, we present how we visually assessed convergence, additional absolute model performance checks and the results of two parameter-recovery studies that confirm that we correctly implemented the PVL-Delta model. For the parameter-recovery studies, we used two synthetic data sets that were generated with the PVL-Delta model. The data-generating parameters correspond to the medians of the individual-level joint posteriors that were obtained by fitting the PVL-Delta model to two real data sets.

Figure A1 shows the HMC chains of one individual-level parameter. From the figure it is evident that the chains have converged successfully from their starting values to their stationary distribution, looking like “hairy caterpillars” that are randomly intermixed. We inspected this type of plot for every parameter to visually assess convergence in addition to the formal diagnostic measure of convergence $\hat{R}$.

Figure A2 presents the fit performance and simulation performance of the PVL-Delta model that was obtained with random draws from the joint posterior distributions over the group-level parameters (hereafter group-level joint posteriors). Note that Figure 9 presents the fit performance and simulation performance based on the individual-level joint posteriors. A comparison of both figures reveals that the fit performance based on the group-level joint posteriors (Figure A2) closely matches the fit performance based on the individual-level joint posteriors (Figure 9). However, there are a few discrepancies in the case of the simulation performance: from Figures 9, A2 it is evident that the simulation performance based on the group-level joint posteriors is more extreme, that is, the most preferred deck is preferred even stronger, whereas the least preferred deck is avoided even stronger. However, it is evident that in general Figure A2 mirrors the conclusion drawn from Figure 9.

Figure A3 presents the results of the first recovery study. This data set contains 18 synthetic participants. The figure contains four panels; each panel illustrates the recovery of one of the four model parameters. In each panel, the mode of the group-level posterior is represented by the dotted line, whereas the solid line represents the true group-level parameter. In addition, the panels can also be used to assess the individual-level recovery: the unfilled dots represent the modes of the individual-level posteriors, whereas the filled dots represent the true individual-level parameters.

Note that the individual-level posterior distributions are not sorted by the subject ID; in order to visualize the degree of individual differences in each model parameter, we sorted the individual-level posterior distributions by the true individual-level parameters.

From Figure A3 it is evident that the group-level updating parameter is slightly underestimated, but the remaining group-level parameters are recovered very accurately. However, the recovery of the individual-level parameters is less accurate. Especially in the case of the shape parameter, most of the individual-level modes differ from the true individual-level parameters by regressing to the mode of the group-level parameter (i.e., shrinkage); small deviations are noticeable in the case of the individual-level loss aversion parameters and the individual-level updating parameter. Yet, in the case of the consistency parameter, most individual-level parameters are recovered very accurately.

Figure A4 presents the results of the second recovery study. This data set contains 30 synthetic participants. It is evident that all group-level parameters are recovered very accurately. However, the recovery of the individual-level parameters is less accurate. Especially in the case of the individual-level shape parameters and the individual-level loss aversion parameters, it is evident that the individual-level modes differ from the true individual-level parameters. Yet, the recovery of the individual-level updating parameters and the individual-level consistency parameters is adequate.
